# Successful use of dalbavancin in the treatment of gram positive blood stream infections: a case series

**DOI:** 10.1186/s12941-022-00507-5

**Published:** 2022-04-26

**Authors:** Connor Evins, Harrison Lancaster, Amanda E. Schnee

**Affiliations:** 1 U of SC School of Medicine Greenville, 607 Grove Road, Greenville, SC 29605 USA; 2 Prisma Health Upstate, 890 W Faris Road, Suite 520, Greenville, SC 29605 USA

**Keywords:** Dalbavancin, Bloodstream, Endocarditis, Outpatient antibiotic

## Abstract

**Background:**

Dalbavancin is a semisynthetic antibiotic used as an alternative to vancomycin for skin infections and osteomyelitis. Its long half-life decreases length of hospitalizations. This study analyzes the effectiveness of Dalbavancin for bacteremia and infective endocarditis.

**Methods:**

The authors performed a retrospective chart analysis on patients who received Dalbavancin due to being poor candidates for PICC placement, poor candidates for prolonged hospitalization, or who were leaving against medical advice. Their hospitalizations were analyzed and results were compiled using descriptive statistics.

**Results:**

Our cohort had 22 patients treated with Dalbavancin for bacteremia and 1 for endocarditis. They were treated with IV antibiotics, typically a regimen of at least vancomycin and a cephalosporin, for a median of 6.5 days prior to receiving Dalbavancin. 20 received one dose, while three received two doses. 22 had confirmed culture clearance and one denied repeat culture. There were no reported side effects from the medication, no readmissions for worsened infection, and no deaths from the infection. 15 patients had follow-up visits within 90 days.

**Conclusions:**

Overall, patients responded well. The lack of readmission to the hospital indicates possible outpatient treatment. This would help decrease cost and comorbidities of long-term hospital stays. These positive results are limited by small sample size and treatment of other antibiotics prior to receiving Dalbavancin. Further research is required to accurately estimate the efficacy of Dalbavancin on bloodstream infections and endocarditis, but these results are promising especially for patients who are not candidates for long term hospitalization or outpatient IV access.

**Supplementary Information:**

The online version contains supplementary material available at 10.1186/s12941-022-00507-5.

## Background

Dalbavancin is a semisynthetic lipoglycopeptide antibiotic which received FDA approval in 2014 for skin and soft tissue infections [1]. It has also been used recently for treatment of osteomyelitis with positive results, although FDA approval has not been given for this indication as of 2020 [2, 3]. Dalbavancin targets gram-positive organisms, most notably methicillin-resistant *Staphylococcus aureus* (MRSA), as it has a mechanism of action that breaks up cell wall synthesis by targeting the terminal d-alanyl-d-alanine residues of the cell wall [1]. In fact, some studies have found it to be superior to vancomycin due to its increased interaction and binding affinity toward this target, which allows for more potent bactericidal activity, particularly towards staphylococcus species [1, 4]. It also boasts a significantly longer half-life allowing for less frequent dosing compared to agents such as vancomycin, giving it an advantage to other IV medications in the outpatient setting [5]. Additionally, Dalbavancin is generally well tolerated, with no reported differences in adverse events in clinical trials vs comparator groups [1].

Due to its long half-life, improved efficacy, and lack of adverse events, Dalbavancin provides an attractive option for treating infections where longer durations of outpatient therapy are required, particularly for patients where continued IV access may represent a challenge. One study demonstrated the concentration of Dalbavancin in cortical bone was above the minimum inhibitory concentration (MIC) for staph aureus for up to 8 weeks [6]. Such concentrations in bone hint towards similar results for other tissues and thus potential efficacy in treating more complicated infections. Given the data that exists for efficacy in osteomyelitis, the drug may be similarly potent against primary bloodstream infections (BSI) or infective endocarditis (IE), both of which often require sustained inpatient IV antibiotic therapy. While significant data exists to support the use of other glycopeptide derivatives to treat BSI or IE, data specifically for Dalbavancin is lacking, though limited retrospective data indicates promise and a recent review summarizes this [7]. Bryson-Cahn et al. reviewed the use of Dalbavancin in intravenous drug users (IVDU) with serious methicillin-sensitive *Staphylococcus aureus* (MSSA) infections and found that over half of the patients demonstrated clinical response [8]. Hidalgo-Tenorio et al looked at the use of Dalbavancin as consolidation therapy for IE or BSI and found that once stabilized, patients had favorable outcomes completing therapy with Dalbavancin [9]. Another study looked at the use of Dalbavancin for more serious infections, though again, limited primarily to patients already stabilized on routine therapy at the start of treatment [10]. The lack of data supporting Dalbavancin is indicative of its infrequent usage for BSI and IE despite its potential advantages to the standard of care.

One area where Dalbavancin is being used off-label for Staphylococcus IE or primary BSI is with patients who are leaving against medical advice (AMA), or in patients who are refusing necessary medical procedures for long term intravenous antibiotic therapy such as a peripherally inserted central catheter (PICC). For severe infections such as endocarditis and bacteremia, especially with gram positive organisms such as MRSA, even full course effective IV treatment with vancomycin or daptomycin have poor outcomes with high mortality rate [11, 12]. Therefore, patients who plan to leave AMA with such infections have even worse outcomes with further increased mortality [13, 14]. Due to its aforementioned long half-life, providing patients with a dose of Dalbavancin prior to leaving AMA reduces the need for a PICC line while also decreasing hospital length. If efficacious, transitioning IV antibiotic care to the outpatient setting in patients who would leave AMA without proper follow up may be a more elegant solution for the increased cost and mortality seen in this population. For skin and soft tissue infections, outpatient antibiotic programs have demonstrated their ability to improve cost by greater than 53% [15].

Given previous retrospective studies showing effectiveness of Dalbavancin, as well as high morbidity and financial implications associated with inpatient antibiotic therapy, this retrospective chart analysis was performed to analyze the effectiveness of Dalbavancin in primary bloodstream infections and infective endocarditis in patients who did not tolerate long term IV antibiotic therapy.

## Methods

We performed a retrospective chart review of patient records from inpatient admissions to the hospital system currently known as Prisma Health in Greenville, SC. The charts were retrieved from a data repository after a request for medical record numbers, date of births, and names for patients in the system between 2014 and 2020 who were prescribed Dalbavancin. These patients were then screened by the investigators for inclusion. The inclusion criteria consisted of age greater than 18 years, and indication for Dalbavancin use being infective endocarditis or bloodstream infection. All other uses for Dalbavancin, including the FDA approved use for skin and soft tissue infections, were not included in our study. IRB approval was received before accessing any patient information.

Data collected includes patient demographics and medical comorbidities such as immunosuppression, diabetes, history of intravenous drug use, or history of similar infections. In regards to the actual indication for Dalbavancin, we recorded initial admission labs and vital signs, length of hospitalization, other antibiotics used during admission, the causative organism, record of culture clearance, and reason for using Dalbavancin versus an FDA approved antibiotic such as refusing medical procedures or threatening to leave against medical advice. The chart was also reviewed for follow up visits with infectious disease, readmissions to hospital, reasons for readmission to hospital, and death within 6 months of discharge. Follow up and mortality data was collected via chart review for office visits with Prisma Health Upstate Infectious Disease and tracked to 90 days. Patients were identified as having follow-up if they attended one office visit within 90 days of their discharge. We also recorded adverse effects of Dalbavancin during initial dose in hospital as well as upon outpatient follow up.

The collected data was analyzed using descriptive statistics to report outcomes of this study. Limited patient identifiers were used during data collection, and all study data was stored on password protected computers with limited access only to investigators. No results or data were released to the public, and no protected patient information was used to describe the results. The patients whose charts were used in this study were never contacted for additional information, and no physical contact with patients was ever needed for this study.

## Results

Of the 128 patients treated at Prisma Health between January of 2014 to 2020 with Dalbavancin, 23 met criteria for our study and were treated for either blood stream infection or infective endocarditis. Of these 23 patients, 22 of these patients were treated for bacteremia and one was treated for tricuspid endocarditis. Nine out of 23 patients received transesophageal echocardiograms (TEE), 12 received transthoracic echocardiograms (TTE), and two patients refused indicated imaging for endocarditis diagnosis. Organisms treated included 6 MRSA, 7 MSSA, 2 *S. epidermidis*, 2 *E. faecalis*, and 3 streptococcal species (Additional file 1: Table S1). Infection sources were varied and included 2 skin, 7 catheter/port/central catheter associated, 3 urine, 1 pulmonary, 4 from IVDU, and 4 unknown (Additional file 1: Table S1). All patients were given IV antibiotics for an average of 10 days prior to being given Dalbavancin. 22 of these patients were culture negative before this transition (Additional file 1: Table S1). The remaining patient refused repeat blood cultures. 19 patients were prescribed a single dose, 17 of which were 1500 mg and 2 were 1125 mg (Additional file 1: Table S1). The 5 remaining patients were prescribed 2 doses at 1500 units at a 7 day interval, however, 2 did not return for their subsequent dose (Additional file 1: Table S1).

Of the 23 patients that met inclusion criteria, 15 (65%) of these patients had significant comorbidities, including 6 (26%) who were immunocompromised (either via medications or neutropenia), 4 (17%) with uncontrolled Type II DM (A1C > 8.5), and 6 (26%) were noted to have a history of IV drug abuse (Additional file 2: Table S2). None of the patients reviewed had either HIV or cirrhosis. 3 (13%) of patients had a history of chronic kidney disease with an average GFR of 13, 2 (9%) required dialysis during hospitalization, and 1 (4%) was on chronic TPN therapy due to malabsorption syndrome. Notably, 11 (46%) of the patients in our study had a previous or current (during the hospital admission that led to Dalbavancin administration) history of leaving AMA or refusing physician recommended care (Additional file 2: Table S2).

Post Dalbavancin infusion, 15 (63%) patients had some sort of follow up within 90 days of their hospital discharge (Additional file 2: Table S2). Of these 15 patients, none were noted to have an adverse event associated with their infusion or at subsequent follow up encounters. The remaining 9 (38%) patients were lost to follow up in 90 days (Additional file 2: Table S2). Of note, two of these patients died from time of hospitalization to data collection. Cause of death for one patient was metastatic gastric carcinoma and occurred within 4 days of discharge. The other patient death was 95 days after discharge, and cause was not directly documented. 9 (39%) patients were readmitted within 30 days of their hospital discharge. None of these readmissions were related to their original infection. However, one patient was admitted within 90 days of original diagnosis due to an intra-abdominal abscess with identical organism.

## Discussion

Patients in our study who received Dalbavancin for infective endocarditis or bloodstream infection had no reported adverse events, complications on follow up, or recorded mortality directly related to their infection. While our cohort was small, this supports previous studies showing Dalbavancin, even for BSI, is both well tolerated and efficacious [1]. Considering the frequency of side effects or adverse events from competing antibiotics, such as acute kidney injury with vancomycin, it is encouraging Dalbavancin is so well tolerated among patients who are severely sick with multiple comorbidities. Our cohort also included a wide variety of bacterial species, sources of infection, and comorbidities, all of which responded well.

A large percentage of our patients were immunocompromised in some way. 26% of patients were receiving some form of immunosuppressive therapy, either in the form of steroids for chronic disease or chemotherapy for cancer treatment. These patients are at significantly increased risk of nosocomial infection, making them ideal candidates for outpatient versus inpatient therapy, particularly when indwelling catheters can be avoided. The source for many of these infections was also long-term IV access in the setting of a surgically placed port. This includes the patients on chemotherapy, and a patient with malabsorption requiring long term TPN therapy. Because of the need to replace ports when they are the source of infection, particularly with organisms like *S. aureus*, these patients potentially would have faced the acquisition of multiple central access sites, thus forcing them to undergo additional procedures with heightened infectious risk in order to complete therapy. Dalbavancin, with its prolonged half-life, was an optimal therapy here as it both expedited discharge as well as limited procedures and central access catheters.

Other patients who were not good candidates for long term antibiotic therapy included one patient where PICC line could not be placed per nursing, and others who had a history of IVDU (Additional file 1: Table S1). As previously stated, there is increased risk for infection or readmission to the hospital for patients with history of IVDU [16]. 6 out of 23 in our cohort had a history of IV drug use and were not given central venous access for this reason. All 6 were treated successfully with Dalbavancin, with no reported readmission or adverse outcome. This is not only beneficial for the health of the patients but is indeed also a positive aspect of therapy aimed at decreasing costs associated with readmission or prolonged hospital stays for inpatient antibiotics.

A significant portion of our cohort, 48%, received Dalbavancin due to refusal of medical treatment or threatening to leave against medical advice (Additional file 2: Table S2). For these patients, Dalbavancin presents an excellent alternative due to its long half-life which enables weekly dosing. Even though these patients have poor follow up data, our cohort did not have any readmissions to the hospital for worsened infection. Additionally, there was no mortality attributable to their infection. This is significant, as many studies have shown patients who leave AMA have a significantly increased rate of readmission or death [13, 14]. Readmission due to inadequate treatment adds tremendous financial strain to our healthcare system, and Dalbavancin provides an attractive option for alleviating this issue [15].

Despite positive data in this review, there remain significant limitations to our study. Most notably, we have an extremely small cohort of 23 patients who qualified during this 6-year time frame. This is expected, due to the treatment not being first line for such severe infections. This lack of sample size and patient variability makes it difficult to accurately assess the treatment. Furthermore, our data only includes readmission and follow-up data associated with our hospital system. It is possible that patients experienced adverse events associated with their original infection or infusion that this study was unable to document. Another challenge with our study is the fact that the majority of our cohort cleared cultures while on a different antibiotic and received an average of 10 days of antibiotics prior to transition to Dalbavancin. Of note, the average days of treatment prior to transition is skewed by two patients who received prior antibiotics for 59 and 36 days respectively, and without including these two outliers the average length of initial antibiotic use decreases to 7 days. Regardless of length of treatment, we still have documented evidence of culture clearance on 22 out of 23 patients prior to transition (Additional file 1: Table S1). To note, though, documentation of clearance is likely due to our patients receiving antibiotic therapy beginning in the emergency department prior to admission, and typically broad-spectrum coverage. While this is presently the standard of care, it limits our ability to fully assess the efficacy of Dalbavancin alone on these infections or as a primary agent for treatment. This is the same challenge seen in previous studies by Hidalgo-Tenorio and Tobudic, where they were unable to accurately assess Dalbavancin’s impact in the vacuum as a first line treatment of gram-positive infective endocarditis or bloodstream infections [9, 10].

## Conclusion

Despite these challenges, the lack of adverse events, readmissions to the hospital for more antibiotics, and excellent results upon follow up with infectious disease outpatient are promising signs of effectiveness. Further studies, in particular a prospective cohort study, are needed to further advance the use of Dalbavancin in these types of infections. However, due to its obvious advantages as a cost-saving outpatient medication with excellent clinical data against gram-positive organisms, its future use as a transitional agent for patients with bacteremia and potentially endocarditis who have cleared cultures is promising.

## Supplementary Information

**Additional file 1: Table S1. **Characteristics of patients treated with dalbavancin

**Additional file 2: Table S2. **Patient demographics

## Data Availability

The datasets used and/or analyzed during the current study are available from the corresponding author on reasonable request.

## Abbreviations

MRSA: Methicillin-resistant *Staphylococcus aureus*
MIC: Minimum inhibitory concentration
BSI: Primary bloodstream infections
IE: Infective endocarditis
IVDU: Intravenous drug users
MSSA: Methicillin-sensitive *Staphylococcus aureus*
AMA: Against medical advice
PICC: Peripherally inserted central catheter
TEE: Transesophageal echocardiograms
TTE: Transthoracic echocardiograms
