# Supplementary material for: Successful use of dalbavancin in the treatment of gram positive blood stream infections: a case series
Source: Ann Clin Microbiol Antimicrob. 2022 Apr 26;21:16. doi: 10.1186/s12941-022-00507-5 (PMC9044886; doi:10.1186/s12941-022-00507-5)
Supplement: Supplementary file 1 — Additional file 1: Table S1. Characteristics of patients treated with dalbavancin [file 12941_2022_507_MOESM1_ESM.docx]

**Table 1: Characteristics of patients treated with dalbavancin**

| **Patient Number** | **Organism** | **Source** | **Duration of antibiotics before Dalbavancin (days)** | **Documented culture clearance** | **Number of Dalbavancin doses** | **Follow up in 90 days** | **Readmission** | **Abx before dalba** |
| --- | --- | --- | --- | --- | --- | --- | --- | --- |
| 1 | *S. agalactiae* | Blood (port) | 15 | Yes | 1 | Yes | Port placement | Vancomycin, daptomycin, ciprofloxacin |
| 2 | MRSA | Blood (IVDU) | 4 | Yes | 1 | No | No | vancomycin |
| 3 | MSSA | Blood (IVDU) | 13 | Yes | 1 | Yes | No | Vancomycin, cefazolin |
| 4 | MSSA | Cellulitis/phlebitis | 4 | Yes | 2 | Yes | No | Nafcillin, vancomycin, ceftriaxone |
| 5 | MSSA | Urine | 3 | Yes | 1 | Yes | No | Ceftriaxone, cefazolin |
| 6 | VGS | Blood | 10 | Yes | 1 | No | No | Vancomycin, ceftriaxone, ciprofloxacin |
| 7 | MSSA | Blood (central catheter) | 6 | Yes | 2 | Yes | Ommaya placement | Ceftriaxone, piperacillin/tazobactam vancomycin, |
| 8 | *S. epidermidis* | Blood (central catheter) | 4 | Yes | 1 | Yes | No | Vancomycin |
| 9 | MRSA | Unknown | 7 | Yes | 1 | Yes | Pneumonia | Vancomycin, levofloxacin |
| 10 | *S. epidermidis* | Blood (central catheter) | 4 | Yes | 1 | Yes | Fistula placement | Vancomycin, ceftriaxone, aztreonam |
| 11 | MRSA | Blood | 10 | Yes | 1 | No | No | Vancomycin, piperacillin/tazobactam |
| 12* | *E. faecalis* | Blood (port) | 7 | Yes | 1 | No | No | Vancomycin, ceftriaxone |
| 13 | MRSA | Unknown (possible cellulitis) | 7 | Yes | 1 | No | Abdominal hematoma | Vancomycin, tobramycin, piperacillin/tazobactam |
| 14 | MSSA | Lung | 6 | No | 1 | No | Gastritis | Vancomycin, cefazolin, piperacillin/tazobactam |
| 15 | MRSA | Unclear (possible pyelonephritis) | 59 | Yes | 1 | No | No | Vancomycin, ceftaroline, gentamicin, piperacillin/tazobactam |
| 16 | *S. pyogenes* | Cellulitis | 4 | Yes | 1 | Yes | No | ceftriaxone |
| 17 | Group G Strep | Unknown (possible skin) | 5 | Yes | 1 | Yes | No | Vancomycin, ceftriaxone, piperacillin/tazobactam |
| 18 | *E. faecalis* | Urine | 8 | Yes | 1 | No | Cystitis with polymicrobial gram negative bacteremia | Vancomycin, ceftriaxone |
| 19** | *S. dysgalactiae* | Blood (catheter) | 2 | Yes | 1 | No | No | Vancomycin, ceftriaxone |
| 20 | MSSA | Blood (catheter) | 6 | Yes | 2 | Yes | Seizures, hyponatremia, MVC | cefazolin |
| 21 | MRSA | Urine | 6 | Yes | 1 | No | UTI | Vancomycin, ceftriaxone |
| 22** | MSSA | Blood (IVDU) | 11 | Yes | 1 | No | No | Vancomycin, cefepime, piperacillin/tazobactam, tobramycin, cefazolin |
| 23 | MRSA | Blood (IVDU, endocarditis) | 36 | Yes | 1 | Yes | No | Ceftriaxone, cefazolin, clindamycin, vancomycin, daptomycin, ceftaroline |

# Key: IVDU = intravenous drug use; VGS = viridens group streptococcus; MVC = motor vehicle collision

# *Patient died within 90 days following discharge

# **Patient did not return for second prescribed dose
