# Supplementary material for: Successful use of dalbavancin in the treatment of gram positive blood stream infections: a case series
Source: Ann Clin Microbiol Antimicrob. 2022 Apr 26;21:16. doi: 10.1186/s12941-022-00507-5 (PMC9044886; doi:10.1186/s12941-022-00507-5)
Supplement: Supplementary file 2 — Additional file 2: Table S2. Patient demographics [file 12941_2022_507_MOESM2_ESM.docx]

# Table 2: Patient demographics

| Average Age (years) |  | 52 (range: 28-94) |
| --- | --- | --- |
| Male |  | 52% (n=12) |
| Female |  | 48% (n=11) |
| Average Weight (kg) |  | 80.5 (range: 50-239) |
| Race/Ethnicity |  |  |
|  | White/Caucasian | 60% (n=14) |
|  | Black/African American | 26% (n=6) |
|  | Hispanic/Latin American | 4% (n=1) |
|  | Asian/South Pacific Islander | 4% (n=1) |
|  | Other | 4% (n=1) |
| Medical history |  |  |
|  | Malignancy | 17% (n=4) |
|  | Organ Transplant | 9% (n=2) |
|  | Uncontrolled Diabetes (A1c>8.5) | 17% (n=4) |
|  | IV Drug Use | 26% (n=6) |
|  | Immunosuppressed (medication related) | 26% (n=6) |
|  | Neutropenia | 9% (n=2) |
|  | Chronic Kidney Disease | 13% (n=3) |
|  | Required Dialysis | 9% (n=2) |
| Average Length of hospitalization (days) |  | 10 |
| Prior similar infection |  | 17% (n=4) |
| Patient death within 90 days |  | 4% (n=1) |
| Patient Death greater than 90 days |  | 4% (n=1) |
| Left AMA/Refused Treatment |  | 48% (n=11) |
